# Diagnostic Accuracy of Pre-Biopsy MRI and CT Features for Predicting Vertebral Biopsy Yield in Suspected Vertebral Discitis Osteomyelitis: A Retrospective Single-Center Study

**DOI:** 10.3390/diagnostics15141760

**Published:** 2025-07-11

**Authors:** Amirmasoud Negarestani, Andrew Pasion, Caleb Bhatnagar, Zuhaib Khokhar, Ashima Kundu, Samantha Diulus, Jorge P. Parada, Emad Allam

**Affiliations:** 1 Department of Radiology, Loyola University Medical Center, 2160 S 1st Ave, Maywood, IL 60153, USA; andrewppasion@gmail.com (A.P.); calebbhatnagar@gmail.com (C.B.); zakhokhar1@gmail.com (Z.K.); ashimak007@gmail.com (A.K.); scdiulus@gmail.com (S.D.); emad.allam@lumc.edu (E.A.); 2 Department of Infectious Diseases, Loyola University Medical Center, 2160 S 1st Ave, Maywood, IL 60153, USA; jparada@lumc.edu

**Keywords:** vertebral discitis osteomyelitis (VDO), spinal biopsy, vertebral biopsy, MRI/CT-based scoring system, spinal osteomyelitis, antibiotic treatment, change in management

## Abstract

**Background/Objectives**: Vertebral discitis osteomyelitis (VDO) is a serious infection involving the vertebral bodies and intervertebral discs, often requiring biopsy for pathogen identification. However, biopsy yields are variable, and guidance on patient selection remains limited. This study aimed to assess how biopsy culture results influence clinical management and to develop imaging-based scoring systems to predict biopsy outcomes. **Methods**: In this retrospective study, 70 patients who underwent image-guided vertebral biopsy for suspected VDO between 2013 and 2022 were reviewed. Pre-biopsy MRI and CT findings were scored using novel, simplified criteria. MRI was graded based on soft tissue involvement, while CT evaluated the presence or absence of a vacuum phenomenon. Culture results were correlated with imaging scores and subsequent changes in antibiotic management. Statistical analysis included logistic regression, ROC analysis, and interobserver agreement using Cohen’s Kappa. **Results**: Of the 70 patients, 27 (38.6%) had positive cultures, and 20 (28.5%) experienced changes in management. Among the 48 patients with both MRI and CT imaging, MRI scores indicating soft tissue involvement and absence of the vacuum sign on CT were independent predictors of positive culture (*p* = 0.022 and *p* = 0.047, respectively). The combined predictive model showed an AUC of 0.76. Interobserver agreement was excellent (κ = 0.90 for MRI, κ = 0.95 for CT). **Conclusions**: MRI and CT features can be used to predict biopsy yield and guide clinical decisions in suspected VDO. These scoring systems may help clinicians identify patients most likely to benefit from biopsy, potentially improving outcomes and minimizing unnecessary procedures.

## 1. Introduction

### 1.1. Background and Clinical Significance

Vertebral discitis osteomyelitis (VDO) is a serious and potentially life-threatening disease affecting the vertebral body, intervertebral discs, and in more advanced stages, the paraspinal and epidural spaces [1,2]. There is an increasing incidence of VDO in the United States. This trend is multifactorial and may be attributed in part to the increased rate of spinal interventions, the increased life expectancy of patients with chronic and immunosuppressive diseases, and the increased prevalence of intravenous drug use [2,3,4]. The clinical presentation of VDO varies from fever and back pain to spinal instability and neurological deficits [5].

### 1.2. Diagnostic Modalities

While VDO can be diagnosed using a variety of imaging modalities, MRI and CT are the most commonly used. Utilizing different sequences such as T1-weighted, fat-saturated T2, STIR, and post-contrast T1 images increases the sensitivity and specificity of MRI to 96% and 92–94%, respectively, making it the preferred imaging modality for diagnosing VDO [6,7,8]. CT can also help diagnose VDO. Signs of VDO on CT include destructive endplate and paraspinal inflammatory changes. On the other hand, the presence of a vacuum sign in the disc space on CT is mainly associated with degenerative joint diseases and trauma [5,9,10].

After initial imaging, further evaluation of VDO often includes image-guided bone biopsy or aspiration. In 2015, the Infectious Diseases Society of America (IDSA) suggested: “Definitive therapy should be based on the result of culture and in vitro susceptibility testing” [11]. Compared to alternative methods of obtaining a tissue sample, image-guided biopsies have lower complication rates, shorter hospital stays, and lower costs. However, reported yield from image-guided biopsy/aspiration has shown variable ranges from 31% to 91%, with two recent meta-analyses showing positive yields of 48% and 52.2% compared to 71–91% positive yields from open biopsies [5,12,13,14]. Some studies tried to evaluate the biopsy yields based on the site of the biopsy and different tissue types. These studies suggested that to increase the yield of a biopsy, the biopsy target should be shifted toward paraspinal or extraosseous involvement [15,16,17,18]. Husseini et al. discussed the controversies surrounding the benefits of targeted antimicrobial therapy versus empiric therapy in patients with high suspicion of VDO but without confirmatory biopsy. After evaluating multiple studies, these authors concluded that the 2015 IDSA guidelines should be followed [5].

Despite the diagnostic utility of MRI and CT, no standardized scoring system exists to predict biopsy culture positivity based on pre-biopsy imaging findings. Existing classifications, such as the AO spine spondylodiscitis classification, the Spinal Infection Treatment Evaluation (SITE) Score, and the Spinal Instability Spondylodiscitis Score (SISS), focus on assessing disease severity, spinal instability, or guiding treatment decisions (e.g., surgical vs. conservative management) rather than predicting biopsy yield [19,20,21]. For example, the SITE Score evaluates neurology, location, radiological changes, pain, and comorbidities to recommend surgical intervention, while the SISS assesses spinal instability based on location, bone lesions, alignment, and pain. However, these systems incorporate subjective or nonspecific findings (e.g., pain, bony destruction) that may overlap with degenerative or neoplastic conditions, reducing their utility for biopsy yield prediction [19,22]. To address this gap, we developed an MRI and CT scoring system that leverages specific imaging features such as soft tissue involvement on MRI and the vacuum phenomenon on CT to predict culture outcomes.

### 1.3. Study Objectives

This retrospective study has two primary objectives: first, to assess the impact of positive biopsy results on clinical management, particularly how culture results guide antibiotic therapy; and second, to predict the likelihood of positive biopsy outcomes in patients with suspected vertebral discitis osteomyelitis (VDO) using pre-biopsy MRI and CT findings. This study aims to improve the understanding of imaging and biopsy roles in VDO diagnosis and treatment, ultimately enhancing patient outcomes through evidence-based decision-making.

## 2. Materials and Methods

### 2.1. Study Design and Patient Selection

**Figure 1:**
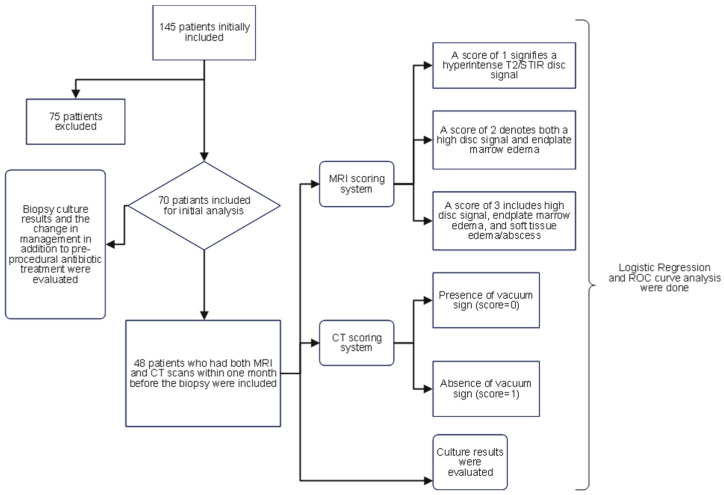
The flowchart illustrates the process of patient selection and the subsequent analysis in this study.

The Institutional Review Board (IRB) of our institution approved this study. The Illuminate database (data retrieval software at our institution) was queried to retrospectively identify patients who underwent image-guided percutaneous vertebral biopsies between January 2013 and April 2022. The following criteria were taken into account when selecting patients: firstly, they had to be 18 years of age or older; secondly, they had to have imaging results suggestive of vertebral osteomyelitis (decreased signal in T1; hyperintense T2/STIR signal in disc or vertebral endplates or paravertebral soft tissue; enhancement in the disc, adjacent vertebral body, and/or soft tissue edema/abscess; erosion or destruction of vertebral endplate) [8,23]; and lastly, a high clinical suspicion of osteomyelitis, such as back pain, fever, and/or neurological deficits upon presentation. The exclusion criteria included cases in which the planned biopsy entry site showed evidence of a pressure ulcer or skin infection, or where biopsy results were positive for malignancy. Initially, 145 patients were identified, 75 of whom were excluded based on these conditions, leaving 70 patients in the initial analysis. To ensure homogeneity in radiological assessment, only patients who had both MRI and CT scans within one month before the biopsy were included in the analysis of pre-biopsy imaging findings and culture outcomes, reducing the sample size to 48 patients. The flowchart (Figure 1) visually presents the patient selection process and the subsequent analysis.

Relevant patient data were comprehensively reviewed from the hospital’s electronic medical record system. Data included baseline patient demographics, laboratory tests, and microbiology results. Clinical data such as age at the time of the procedure, gender, the date of the procedure, the biopsy level, blood cultures, and type of organism were collected. Additionally, all oral and intravenous antibiotic regimens were recorded before and after the biopsy procedure. All vertebral biopsies were performed under CT guidance, either by a radiology attending physician or by a radiology resident under the direct supervision of an attending physician. All biopsy samples were evaluated for aerobic and anaerobic cultures, and, in select cases, for acid-fast bacilli and fungal organisms when clinically indicated. A change in management was defined as any modification to the antimicrobial regimen following the availability of culture results. This included narrowing broad-spectrum empiric therapy, initiating treatment in previously untreated patients, or adjusting therapy based on the susceptibility profile of the identified organism. Specifically, tailoring the antibiotic regimen to target the organism isolated from the biopsy was considered a direct change in clinical management, provided the organism was not concurrently isolated from another source, such as blood cultures [24].

One musculoskeletal imaging specialist and one general radiologist, each with at least four years of practice experience, assessed the patients’ imaging from the electronic picture archiving and communication system (PACS) and objectively quantified the pre-procedural imaging findings. Each radiologist independently interpreted the patients’ imaging studies and assigned a score to each patient. Radiologists who interpreted CT and MRI scans were blinded to biopsy results to minimize diagnostic bias. To ensure an unbiased analysis of diagnostic accuracy, clinicians interpreting the biopsy results were also blinded to the imaging findings.

### 2.2. Imaging Scoring Systems

MRI and CT are widely used to evaluate vertebral osteomyelitis [5,25]. MRI detects signal changes in the intervertebral disc, vertebral body, and paravertebral soft tissues, while CT identifies bone erosion, abscess formation, soft tissue swelling, and disc involvement. However, CT findings are nonspecific for intervertebral disk space narrowing and may underestimate the soft tissue involvement [11,22]. Despite efforts to utilize these imaging findings in patient management, their role remains controversial [5,12]. Therefore, the authors of the present study developed an MRI and CT scoring system in an attempt to improve the decision-making process and provide insight into identifying suitable candidates for spinal biopsy who may benefit the most from the invasive process of vertebral biopsy.

**Figure 2:**
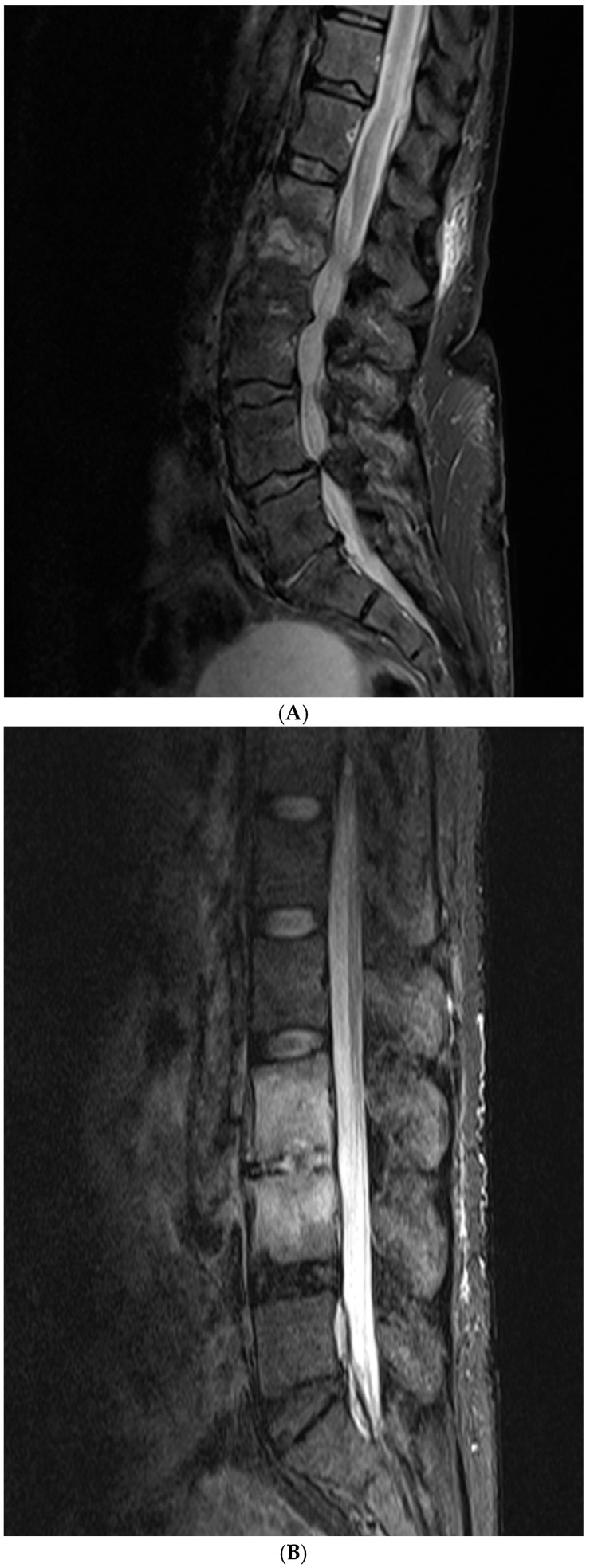

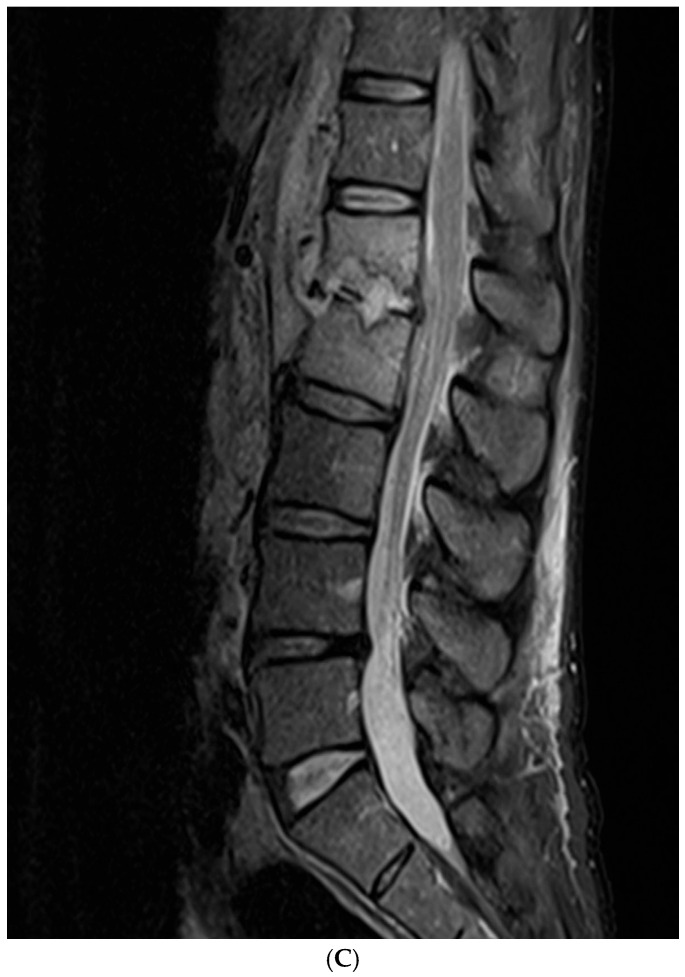
(**A**) Score of 1 indicates hyperintense T2/STIR signal in the disc; (**B**) score of 2 indicates hyperintense T2/STIR signal in the disc and marrow edema in vertebrae; (**C**) score of 3 indicates hyperintense T2/STIR signal in the disc, marrow edema in vertebrae, and soft tissue edema and/or abscess.

The MRI scoring system utilizes a scale from 1 to 3, with each score representing distinct radiological findings. A score of 1 signifies a hyperintense T2/STIR disc signal, suggesting possible inflammation and edema related to early infection (Figure 2A). Moving up the scale, a score of 2 denotes both a high disc signal and endplate marrow edema, likely indicative of more advanced stages of VDO (Figure 2B). Finally, a score of 3 includes high disc signal, endplate marrow edema, and soft tissue edema/abscess, suggesting severe involvement (Figure 2C). It should be noted that all scoring assessments above were verified using other MRI sequences to improve classification accuracy. For instance, soft tissue edema and abscess can be distinguished from one another using MRI with IV contrast [5]. However, differentiating edema from abscess was not necessary for the scope of this study because the presence of either entity could be interpreted as more severe imaging findings related to VDO. Also, as 14 out of the 48 patients (29%) did not have an MRI with contrast before the biopsy, this approach enabled us to include more patients in our study and simplify the scoring system for greater usability. Thus, we included both soft tissue edema and abscess in the same category, denoted by a score of 3.

Regarding the CT scoring system, while CT may show destructive endplate changes in VDO along with inflammatory changes or fluid collection, the equivocal findings may put the clinician at risk for a diagnostic misinterpretation [22]. Additionally, the diagnostic performance of CT has not been systematically evaluated [5,26]. On the other hand, vacuum phenomena, defined as the presence of gas in the intervertebral disc, is mostly known as a sign of the presence of degenerative disc disease, with a specificity and sensitivity of 89.7% and 89.3%, respectively [27]; however, this sign could also be presented in the patients with VDO with a frequency rate of 20.4% in culture-positive patients [28]. Overall, we decided to incorporate the vacuum sign to constitute our scoring system as it supports the existence of degenerative joint disease over VDO. Thus, the presence or absence of the vacuum sign at the biopsy level was assessed using patients’ pre-procedural CT scans, with a score of 0 indicating the presence of the sign and a score of 1 indicating its absence. Power calculations were conducted to evaluate the statistical robustness of the study’s analyses.

Both CT and MRI scans used for the above scoring system were obtained within one month before the biopsy to ensure the relevance and accuracy of the radiological findings.

In this study, CT scans were performed using a 64-slice CT scanner with a tube voltage of 120–140 kVp, a slice thickness of less than 3 mm, and bone and soft tissue reconstruction algorithms. Images were reviewed in both bone and soft tissue windows. MRI scans were performed with a 1.5 T MRI system using standard sequences such as T1-weighted, T2-weighted, STIR, and post-contrast T1-weighted images when appropriate. The slice thickness was set to 3 mm, with a field of view (FOV) of 120–200 mm. Both the sagittal and axial planes were evaluated. In patients who did not have an MRI with contrast, paravertebral soft tissue involvement was evaluated based on T2/STIR signal intensities.

### 2.3. Statistical Analysis

Statistical analysis was performed using IBM SPSS 23.0 software (IBM Corp., Armonk, NY, USA). Data were appropriately described in the form of means and standard deviations (SDs) for continuous variables or frequency distributions (percentages) for categorical variables. An independent sample *t*-test and chi-square test were used to analyze group differences and categorical associations. The relationships between the variables were analyzed using cross-tabulation, and sensitivity and specificity were computed accordingly. Logistic regressions were performed to identify independent predictors of sample culture outcomes. These regression models incorporated culture results, MRI, and CT findings to assess their influence on biopsy outcomes. Additionally, receiver operating characteristic (ROC) curve analysis was used to evaluate the discriminative power of the combination of CT and MRI scores in terms of predicting culture results. The ROC curve and the Mann–Whitney-U test were also used to compare the predicted probability of combined CT and MRI scores between the groups with positive and negative culture results. Cohen’s Kappa assessed interobserver reliability and agreement for the CT and MRI scoring systems.

Power analysis was performed to determine the robustness of the finding and the ability to detect significant differences in key parameter comparisons. To ensure compliance with the study’s statistical framework, all power calculations were carried out using Python (Version 3.12.10). For chi-square analyses of the CT vacuum sign and MRI scoring system, effect sizes (Cohen’s h) were computed using the proportions of positive findings in each group. Power was determined using the total sample size (*n* = 48) and a two-sided significance level (α = 0.05). The predictive performance of the logistic regression model was assessed using the area under the curve (AUC), which was converted to an approximate effect size (d) using the formula [d = $\sqrt{2}$ Φ^−^^1^(AUC)], where Φ^−1^ is the inverse of the cumulative distribution function of the standard normal distribution. The standardized effect size and the critical z-value for a two-tailed test were compared to determine the power for logistic regression.

## 3. Results

### 3.1. Patient Demographics and Clinical Characteristics

A total of 145 vertebral biopsies were performed between January 2013 and April 2022. However, due to the exclusion criteria, 75 of these biopsies were not included in the analysis. As a result, 70 patients who had image-guided bone biopsy procedures for possible vertebral osteomyelitis were included in the analysis. The average age of the 70 patients who were part of this study was 61 ± 15 years. Of them, 26 (37%) were female and 44 (63%) were male. The majority of patients (94.3%) experienced back pain, while only five patients (7.1%) had a fever. Notably, 64 and 65 patients had inflammatory markers, ESR, and CRP checked. A comparison of the culture-positive and culture-negative groups of patients revealed that the former had a mean ESR and CRP of 68.9 mm/h and 37.2 mg/dL, respectively, with standard deviations of 31.11 and 57.36, and the latter 58.01 mm/h and 17.53 mg/dL, with standard deviations of 34.51. and 26.34, in that order. There was no significant difference between the patient groups with positive and negative cultures according to an independent-sample *t*-test analysis of ESR and CRP. For ESR, the T-statistic was 0.145, and the *p*-value was 0.885; for CRP, the T-statistic was −1.025, and the *p*-value was 0.314.

**Figure 3:**
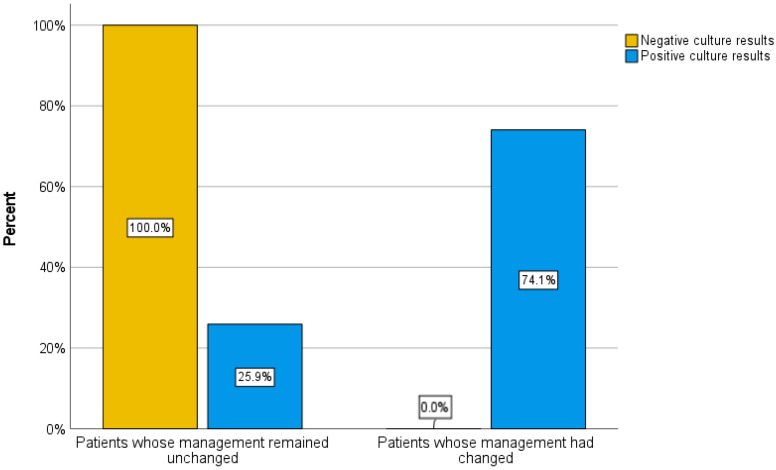
Comparison of changes in management between patients with positive and negative culture results. Patients with positive culture results experienced significant management changes, whereas patients with negative culture results experienced no change in management.

Of the 70 patients, 43 (61.4%) had negative culture results, and 27 (38.6%) had organism growth in their sample culture. Additionally, 20 patients (28.5%) had a change in management based on culture results, whereas 49 patients (71.5%) had no change in management. To describe the association between sample culture results and the presence or absence of a change in patient management, a cross-tab analysis was performed. Among those with positive culture results, 20 patients had a change in management (74.1%), and 7 patients (25.9%) had no change in management. On the other hand, among those who had negative culture results, all 43 patients (100%) experienced no change in management based on culture results by definition (Pearson chi-square: 44.593, degree of freedom = 1, *p* < 0.001) (Figure 3).

Overall, 13 patients (18.6%) received pre-biopsy antibiotic therapy, while 57 patients (81.4%) did not. Among those with negative culture results, 10 patients (23.3%) had a history of pre-biopsy antibiotic treatment, compared to 3 patients (11.1%) with positive culture results who had a history of pre-biopsy antibiotic treatment (Fisher’s exact test, *p* = 0.344). The odds ratio (OR) comparing the likelihood of having a positive culture result between the groups administered no pre-biopsy antibiotics and pre-biopsy antibiotics was 2.42.

### 3.2. Analyses of Imaging Findings

**Figure 4:**
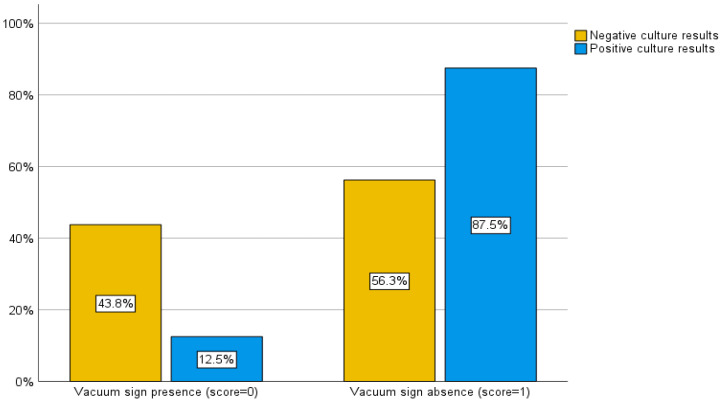
Correlation between CT scan grading system and culture results. The majority of positive culture results (87.5%) were observed in patients in whom the vacuum sign was absent on CT imaging.

Initially, we evaluated the yield of culture results for all patients who underwent spinal biopsies, regardless of the type of advanced imaging that had been carried out beforehand. However, when we analyzed the scoring system, we only included the patients who had both CT and MRI scans performed within a month of the procedure, as some patients had not had both. As a result, 48 patients were evaluated in the subsequent scoring system. In patients whose culture results were negative, a vacuum sign was present in 14 cases (43.8%) and absent in 18 cases (56.3%). In contrast, a vacuum sign was present in 2 (12.5%) and absent in 14 (87.5%) of the patients with positive culture results (Figure 4). The sensitivity and specificity of the CT vacuum sign for our sample in distinguishing positive from negative biopsy culture results were 87.5% and 43.8%, respectively (Pearson chi-square, *p* = 0.03 and likelihood ratio of 5.189).

Additionally, to evaluate the potential confounding effect of age on CT findings, we assessed the correlation between patient age and CT binary scores. The Pearson correlation coefficient was −0.204 (*p* = 0.127), indicating a non-significant trend toward increased likelihood of vacuum sign presence in older patients.

**Figure 5:**
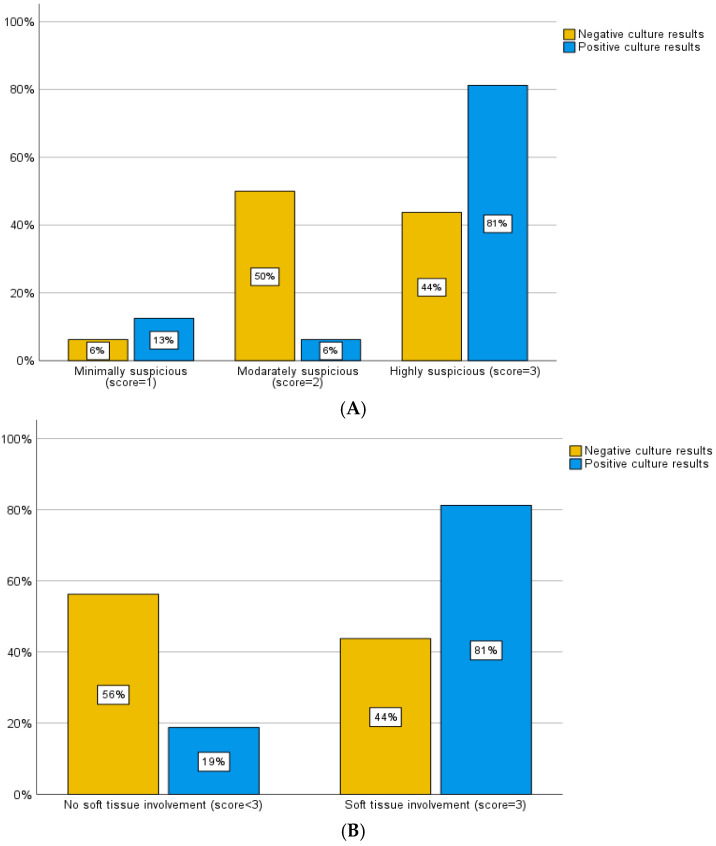
(**A**) Distribution of culture results across different MRI scores. The percentage of positive culture results increased with the highest MRI score; (**B**) comparison of culture results and MRI scores based on soft tissue involvement. Patients with soft tissue involvement (score = 3) had a higher percentage of positive culture results (81%) compared to patients without soft tissue involvement.

The MRI scoring system employed by the present study also evaluated 48 patients who had an MRI within one month of their biopsy. Of the patients with negative biopsy culture results, 2 cases (6.3%) were classified as mild, 16 (50%) as moderate, and 14 (43.8%) as severe by the MRI scoring system. Within the group with positive biopsy culture results, there were 2 (12.7%) cases classified as mild, 1 (6.3%) as moderate, and 13 (81.3%) as severe by MRI criteria (Figure 5A). To enhance the clarity and analysis of these data according to the literature review [5,12], we considered a score of 3 (severe) to be highly predictive of positive biopsy culture results/impact on clinical management and a score of 1 or 2 (mild and moderate) to be cases in which a biopsy was unlikely to alter the outcome. Of the patients whose cultures came back negative, 14 (43.8%) had soft tissue involvement, while 18 (56.3%) did not. Alternatively, of the patients whose cultures were positive, 13 (81.3%) had soft tissue involvement, and 3 (18.8%) did not (Figure 5B). The sensitivity and specificity associated with this classification were calculated to be 81.3% and 56.3%, respectively (Pearson chi-square, *p* = 0.014 and likelihood ratio of 6.488).

Cohen’s Kappa was used to evaluate the interobserver reliability of the MRI and CT scoring systems. The weighted Kappa of 0.901, calculated with quadratic weights, demonstrated nearly perfect agreement between the two radiologists when evaluating MRI scores. The radiologists’ nearly perfect agreement on CT scores was indicated by the unweighted Kappa of 0.954 (Table 1).

**Figure 6:**
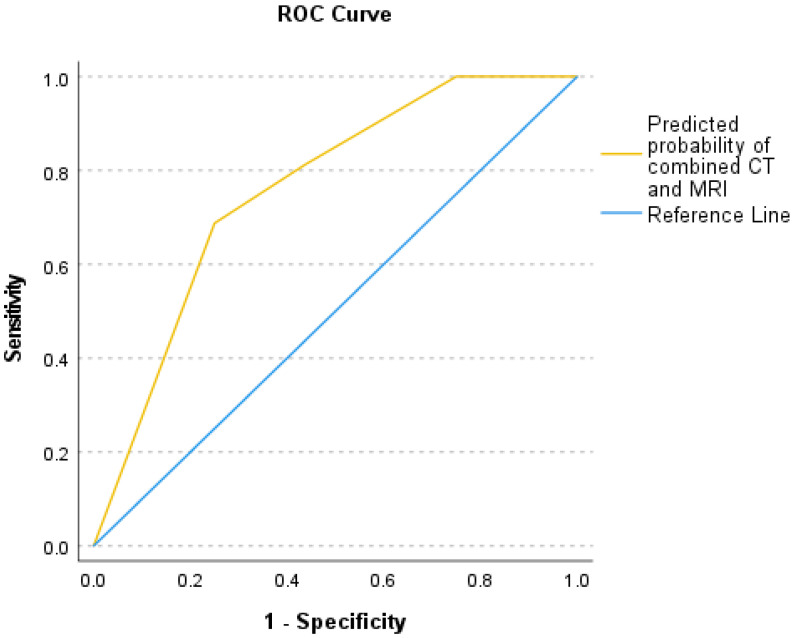
ROC curve comparing the predicted probability of combined CT and MRI for diagnostic accuracy. The yellow line represents the performance of the combined CT and MRI predictions, while the blue reference line represents a model with no discriminative ability. The curve demonstrates the sensitivity and specificity of the combined imaging modalities in predicting outcomes.

Logistic regression analysis examined the relationship between sample culture results as the dependent variable and soft tissue involvement on MRI and vacuum sign on CT as two independent variables that showed a positive coefficient for the MRI scoring system [coefficient B = 1.754, *p*-value = 0.022, and Exp(B) = 5.777] and the CT vacuum sign [coefficient B = 1.738, *p*-value = 0.047, and Exp(B) = 5.685]. CT vacuum sign and MRI soft tissue involvement were relatively independent, according to the correlation matrix (r = 0.01). The area under the curve (AUC) was 0.76, which indicates that this model has a moderate predictive ability (Figure 6). The predicted probability of combined CT and MRI scores was compared between the groups with negative and positive culture results using the Mann–Whitney U test (*p*-value = 0.002). Power calculations demonstrated 99.9% power for the CT vacuum sign, 99.98% for MRI scoring, and nearly 100% for logistic regression, reflecting the robustness of the analyses.

## 4. Discussion

### 4.1. Overview

Vertebral osteomyelitis represents a complex and challenging clinical condition with significant implications for patient morbidity and mortality. The role of image-guided bone biopsy in the diagnosis and subsequent management of this condition has been the subject of ongoing investigations [5,12,14,29]. Direct biopsy is a common secondary diagnostic approach when clinical findings indicate VDO but blood cultures are negative. The identification of the microorganism helps confirm the diagnosis of VDO and customize antibiotic therapy. In the literature, the reported sensitivity of biopsy has varied greatly, ranging from 31% to 91%. Meta-analysis performed to evaluate the effect of prior antimicrobial exposure on sample culture results is hard to understand and apply to clinical practice due to the variability of antecedent antimicrobial exposure definitions used in those studies. Overall, there is little evidence that antecedent antimicrobial therapy affects the microbiologic yield from biopsy [5,30,31,32]. The guidelines of the IDSA suggest treating empirically in cases of positive blood cultures and recommending percutaneous biopsy in cases of suspected VDO without a positive blood culture. They also advise repeating the image-guided biopsy or moving forward with surgical biopsy to improve sensitivity in cases where the percutaneous biopsy yields negative results [5,11,12].

Traditional diagnostic techniques often rely, to a large extent, on biopsy results and qualitative interpretation of imaging findings, as indicated in guidelines like the IDSA. These are usually influenced by variability in biopsy yields and the risk of inconclusive procedures. The current investigation combines MRI and CT features, introducing a predictive framework that quantifies imaging findings and integrates them with clinical parameters. This model compares to the common approach in giving a more objective and non-invasive tool for the prediction of biopsy outcomes. It aims to bridge a major diagnostic gap in current guidelines.

### 4.2. Imaging Findings

In this retrospective study of 70 patients who underwent image-guided bone biopsy for suspected vertebral discitis osteomyelitis (VDO), culture results were negative in 43 cases (61.4%) and positive in 27 cases (38.6%). There was no statistically significant difference in the rate of positive cultures between patients who received antibiotics prior to the biopsy and those who did not. Although an odds ratio of 2.42 suggested a trend toward a higher yield in patients without pre-biopsy antibiotics, this association did not reach statistical significance. These findings are consistent with prior studies by Schiro et al., Hoang et al., and Malik et al., which also reported no significant impact of pre-biopsy antimicrobial therapy on culture positivity rates [30,31,32].

Despite the majority of biopsies being culture-negative, our analysis demonstrated that 74.1% of patients with positive culture results experienced a change in management. In our cohort, culture results guided clinicians to tailor treatment by de-escalating from broad-spectrum empiric regimens, such as vancomycin with ceftriaxone, to more focused antibiotics based on susceptibility data. In some cases, patients who were not receiving antibiotics at the time of biopsy were initiated on appropriate targeted therapy following culture identification. In other instances, results prompted a change in regimen to optimize pathogen-specific coverage. This real-world variability reflects how culture data play a key role in antimicrobial stewardship by enabling more individualized and effective treatment decisions in suspected vertebral discitis osteomyelitis.

We retrospectively evaluated patients’ preprocedural imaging to examine the potential predictive value of CT and MRI scoring systems in achieving positive culture results. Using our developed scoring systems for MRI and CT, we found that a high MRI score, driven by features such as soft tissue edema or abscess, was significantly associated with positive culture results. Conversely, the presence of a vacuum phenomenon on CT was significantly associated with negative culture results. Sensitivity and specificity of the scoring systems were 87.5% and 43.8% for CT, and 81.3% and 56.3% for MRI, respectively. The relatively high sensitivity and moderate specificity of both models suggest potential utility as screening tools to guide biopsy decision-making. It is worth noticing that the non-linear distribution of positive culture rates across MRI scores, with a lower rate in the moderate (score 2, 6.3%) compared to the mild (score 1, 12.7%) group, may be attributed to the small sample sizes in these subgroups. This variability limits the reliability of proportional comparisons for intermediate scores. The MRI scoring system in our study was mainly designed to prioritize severe findings (score 3, 81.3% positive cultures) as predictors of positive biopsy yield, and larger studies are needed to validate the consistency of mild and moderate score predictions.

Using logistic regression and ROC curve analysis, we demonstrated that both the MRI soft tissue score and the CT vacuum sign were statistically significant independent predictors of culture outcomes. The combined model yielded an AUC of 0.76, indicating moderate discriminative performance in identifying patients likely to have positive culture results.

In our study, three patients had concordant blood and biopsy culture results, two with methicillin-resistant Staphylococcus aureus and one with Enterobacter cloacae. However, in all three cases, biopsy results did not lead to any change in clinical management. In retrospect, these biopsies may have been unnecessary, suggesting that in select cases with definitive blood culture findings and characteristic imaging features, biopsy may not always provide additional clinical value. Four additional patients with positive biopsy cultures also experienced no documented change in management, as appropriate antibiotic therapy had already been initiated before the biopsy based on clinical suspicion.

This observation aligns with existing research, which supports that in patients with classic radiologic and clinical signs of vertebral discitis osteomyelitis (VDO) and blood cultures positive for common pathogens such as Staphylococcus aureus or Pseudomonas aeruginosa, the causative organisms of spinal infection are often the same [11,24]. Furthermore, the optimal timing of biopsy in suspected VDO remains debated, particularly whether withholding antibiotics prior to the procedure improves culture yield, and how best to proceed when blood cultures are negative or pending. These uncertainties highlight the need for an individualized, evidence-based approach. However, our findings, consistent with recent studies, suggest that prior antibiotic therapy does not significantly impact the likelihood of obtaining a positive culture result [5,30,31,32].

### 4.3. Clinical Implications

We propose that the introduced scoring system be utilized as a clinical decision-making tool when evaluating patients for spinal biopsy. By applying this model, primary physicians may better anticipate the likelihood of obtaining a positive culture result and adjust management strategies accordingly. For example, in patients with soft tissue involvement on MRI and absence of a vacuum sign on CT, features associated with a higher probability of positive culture, clinicians might consider initiating antibiotic therapy earlier in the disease course, even before the biopsy procedure, or selecting antibiotics with superior tissue penetration. Conversely, in patients with imaging findings associated with negative cultures from biopsy, unnecessary early antibiotic treatment might be avoided.

This targeted approach is particularly relevant given the well-documented challenges of treating vertebral osteomyelitis, where poor bone vascularity can hinder effective antimicrobial delivery. Furthermore, abscess formation and surrounding edema have been shown to further compromise antibiotic penetration to the site of infection [33,34,35]. While early antibiotic administration raises concerns about potentially reducing culture yield, our findings, consistent with prior studies [5,30,31,32], suggest that antecedent antimicrobial therapy does not significantly impact microbiologic yield from biopsy. In non-septic patients with imaging findings strongly suggestive of VDO (e.g., MRI score 3), initiating tissue-penetrating antibiotics before biopsy may be justified to address the infection promptly, given the barriers to antimicrobial delivery in vertebral tissue. However, in the absence of sepsis or systemic compromise, clinical judgment, ideally guided by infectious disease specialists, should balance the need for timely treatment with the preservation of diagnostic accuracy through biopsy. This nuanced approach ensures that patients with high-probability imaging findings receive early intervention while avoiding unnecessary antibiotics in cases where biopsy is unlikely to yield positive cultures.

Incorporating imaging-based predictive tools into the clinical workflow may therefore enhance diagnostic predictability, optimize therapeutic decision-making, and reduce the risks associated with both under- and over-treatment.

### 4.4. Limitations and Future Directions

This study has several limitations. First, its retrospective design introduces potential selection bias and limits causal inference. Second, interpretation of the CT vacuum sign and MRI findings—particularly paravertebral soft tissue involvement—may be subject to inter- and intraobserver variability in borderline cases, despite strong interobserver agreement (Cohen’s Kappa: 0.901 for MRI, 0.954 for CT). Third, the relatively small sample size (*n* = 70 overall; *n* = 48 for imaging analysis) may have reduced the statistical power to detect significant associations, particularly in the case of pre-biopsy antibiotic exposure (OR 2.42; 95% CI: 0.60–9.76). Fourth, to maintain objectivity and simplicity, the CT scoring system focused solely on the vacuum phenomenon, potentially omitting other informative features such as bony destruction, which are included in the AO Spine spondylodiscitis classification. While such features could improve prognostic stratification, they may also introduce ambiguity due to overlap with degenerative or neoplastic conditions. Fifth, 14 of the 48 patients (29%) underwent MRI without contrast, which may have limited the detection of paravertebral soft tissue involvement and affected the accuracy of MRI scoring. Finally, as a single-center study, the findings may not be generalizable to broader populations and clinical settings.

These findings should be considered within specific clinical contexts and their implications for patient care. Future studies should address these limitations by employing prospective, multi-center designs with larger sample sizes to validate the scoring system’s predictive accuracy. Incorporating additional CT findings, such as bony destruction graded by the AO Spine classification, and exploring advanced imaging techniques could refine the model while maintaining specificity for biopsy yield prediction. These efforts will strengthen the scoring system’s clinical utility and support evidence-based decision-making in VDO management.

## Figures and Tables

**Table 1:** Kappa values of inter-observer variability.

	MSK Radiologist (Number of Cases)	General Radiologist (Number of Cases)	Unweighted Cohen’s Kappa	Weighted Kappa (Quadratic)	95% CI for Weighted Kappa
MRI scoring system					
Minimally suspicious	4	4	0.855 ^T^	0.914 ^T^	(0.778–0.978) ^T^
Moderately suspicious	19	21			
Highly suspicious	39	37			
CT scoring system					
Absence of vacuum sign	36	35	0.951 ^†^	0.901 ^†^	(0.859–1) ^†^
Presence of vacuum sign	21	22			

^T^—Measurement agreement between the two radiologists for the MRI scoring system; ^†^—Measurement agreement between the two radiologists for the CT scoring system.

## Data Availability

The data presented in this study are available upon reasonable request from the corresponding author due to institutional policy regarding Protected Health Information.

## Abbreviations

The following abbreviations are used in this manuscript:

VDO	Vertebral discitis osteomyelitis
